# Influence of Perceived Helicopter Parenting, Critical Thinking Disposition, Cognitive Ability, and Learning Motivation on Learning Behavior among Nursing Students

**DOI:** 10.3390/ijerph18031362

**Published:** 2021-02-02

**Authors:** Hyunjoo Oh, Haeryun Cho, So Youn Yim

**Affiliations:** 1 Department of Nursing, Ewha Womans University Medical Center, Seoul 07985, Korea; ewcc@naver.com; 2 Department of Nursing, Wonkwang University, Iksan 54538, Korea; 3 Department of Nursing, Baekseok University, Cheonan 31065, Korea; ysybest@bu.ac.kr

**Keywords:** helicopter parenting, critical thinking disposition, learning motivation, learning behavior, nursing students

## Abstract

The purpose of this study was to determine the influence of perceived helicopter parenting, critical thinking disposition, cognitive ability, and learning motivation on learning behavior in nursing students in South Korea. The participants in this study were 149 sophomore nursing students from two universities using convenience sampling. The two universities were similar in terms of type, curricula, and size. Data were collected from October to November 2017 using self-reported questionnaires. The collected data were analyzed using descriptive statistics, Pearson correlation coefficients, and hierarchical multiple regression with SPSS 22.0. The mean score of perceived helicopter parenting was 3.06 ± 0.65 out of six points. The levels of critical thinking disposition, cognitive ability, learning motivation, and learning behavior were medium. Factors affecting learning behavior were learning motivation (β = 0.40, *p* < 0.001), cognitive ability (β = 0.26, *p* = 0.001), and critical thinking disposition (β = 0.25, *p* = 0.001). These variables explained 32% of the variance in learning behavior (F = 18.21, *p* < 0.001). Teaching methods are necessary to increase the critical thinking disposition and learning competence of nursing students. In addition, it is important to consider the learning motivation of nursing students for effective learning.

## 1. Introduction

The learning competencies of university students include cognitive ability, learning motivation, and learning behavior [1]. Learning behavior is the management of cognitive skills to organize and remember new information, trans-cognitive skills that control learning-related behaviors, and environments that are conducive to learning [1]. As a result, that nursing education involves theoretical education alongside practical education in order to cultivate competent professional nurses who can solve various health problems of patients [2,3], students’ perceived burden of schoolwork may be high. In addition, efficient learning behaviors of nursing students can be considered to be an important factor for effective nursing education because they have to prepare for national exams to obtain a nurse’s license.

In recent years, the number of parents with overprotective parenting attitudes has increased, creating a problem as many parents of college students who are classified as adults pursue a helicopter parenting style [3,4,5,6,7]. Helicopter parenting is an approach in which parents actively intervene in their child’s overall life even after the child becomes an adult, and, in order to help the child, they sometimes engage in excessive intervention that violates the independence and autonomy of the child [8]. In other words, helicopter parents hover around their children and solve their problems [9]. It has been reported that children of helicopter parents have low self-efficacy even after they become adults and have a tendency to depend on parents, and this parenting style also negatively affects their school activities [7,10,11]. However, research on the relationship between helicopter parenting and children’s learning behavior is not enough, so studies to confirm this relationship are needed.

Critical thinking refers to reflective and rational thinking that determines an individual’s beliefs or actions and shapes one’s thoughts and knowledge based on his/her experiences and observations [12]. Critical thinking disposition refers to the tendency toward this thinking, and the motive or desire of critical thinking is problem solving and decision-making [13]. Nursing education deals with critical thinking as this skill is important to improve the ability of students to analyze health problems and apply professional nursing knowledge and skills to solve them [14]. Furthermore, it has been reported that nursing students with a high level of critical thinking disposition have high self-directed learning ability [15]. Therefore, critical thinking disposition is considered to be not only an important competency in the nursing curriculum, but also an important factor for effective learning.

Cognitive ability refers to the ability to acquire knowledge and process information [1]. It is a concept that includes knowledge, understanding, thinking, creativity, and problem-solving ability. In addition, it has been reported to be closely related to academic achievement and self-directed learning ability [1]. Thus, promoting the cognitive ability of college students can improve learning ability and academic achievement. However, studies on cognitive abilities of nursing students are insufficient. Some previous studies dealing with problem-solving abilities, which is a similar concept, reported that nursing students with high problem-solving abilities have a high level of critical thinking disposition [14,16]. Therefore, in order to prepare effective learning strategies for nursing students, it is necessary to understand the relationship between cognitive ability and learning behavior.

Learning motivation is a psychological process that induces and maintains learning by determining the direction, level, and intensity of learning [1]. Nursing students who are highly motivated to learn have a lot of interest in learning, and they not only constantly strive to achieve specific goals, but also have good control over the obstacles to learning [17]. Therefore, several previous studies have considered learning motivation of university students to be an important factor influencing learning behavior [1,17,18].

Recently, with interest in helicopter parenting growing in various academic fields, several studies have examined the relationship between helicopter parenting and depression in undergraduate students [4,19,20], the relationship between helicopter parenting and university students’ academic achievement [6,7], and helicopter parenting attributes [21]. Studies of nursing students have been conducted to conceptually analyze helicopter parenting [9], develop a helicopter parenting scale [22], and examine the relationship between helicopter parenting and learning competence of nursing students [23]. On the other hand, looking at previous research that analyzed the learning competencies of nursing students, it can be seen that studies dealing with learning motivation have been actively conducted [17,18,24,25], but studies dealing with cognitive abilities and learning behaviors of nursing students are inadequate. Moreover, studies examining the relationship between helicopter parenting, critical thinking tendency, cognitive ability, learning motivation, and learning behavior in nursing students are also insufficient.

Therefore, the aim of this study was to understand the effects of helicopter parenting, critical thinking disposition, cognitive ability, and learning motivation on nursing students’ learning behavior. The specific objectives of this study were as follows: (1) Identify the general characteristics of the participants; (2) identify the degree of helicopter parenting, critical thinking disposition, cognitive ability, learning motivation, and learning behavior of the participants; (3) identify the correlation between helicopter parenting, critical thinking disposition, cognitive ability, learning motivation, and learning behavior; and (4) identify the effects of helicopter parenting, critical thinking disposition, cognitive ability, and learning motivation on learning behavior. It is expected that this study will provide the basic data necessary to develop effective learning strategies by grasping the characteristics of nursing students raised by helicopter parents and their learning competence.

## 2. Methods

### 2.1. Study Design

This cross-sectional descriptive correlational study was designed to investigate the potential effects of helicopter parenting, critical thinking disposition, cognitive ability, and learning motivation on learning behavior among nursing students.

### 2.2. Participants

**Figure 1:**
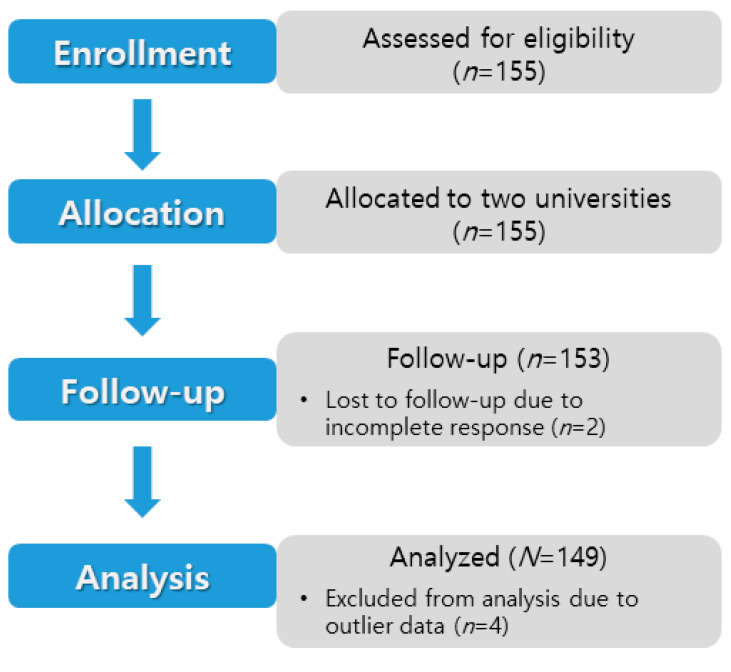
Flow diagram of data collection in this study.

The participants of this study were second-year students enrolled in the departments of nursing at two universities, one located in Chungcheong Province and the other in Jeolla Province. The two universities were similar with respect to curricula, total number of students, and type (four-year university). Both were certified by Korean Accreditation Board of Nursing Education. The sample size was calculated using the G*Power 3.1.3 program [26], and the number of samples required for the median effect size, significance level of 0.05, power of 0.80, and four predictors was 85. In consideration of dropouts, questionnaires were distributed to 155 subjects. Participants were recruited—77 nursing students in Chungcheong Province and 78 nursing students in Jeolla Province—using convenience sampling. There were no unrecovered responses. Two questionnaires with many unanswered items were dropped, and four responses corresponding to outliers in the normality review were dropped. Finally, 149 responses were analyzed (Figure 1).

### 2.3. Measurement

#### 2.3.1. Helicopter Parenting

The instrument used to measure the degree of helicopter parenting was the Korean version of the helicopter parenting scale validated by Chae et al. [22]. This scale revised the Helicopter Parenting and Autonomy Supportive Behaviors tool developed for undergraduate students by Schiffrin et al. [27]. It consists of 26 questions including autonomy support, daily life hovering, school life involvement, career decision-making involvement, serve, and taste involvement. Each question is measured on a 6-point Likert scale from 1 (completely disagree) to 6 (completely agree). The measurable score ranges from 1 to 6. The higher the score, the higher the helicopter parenting level. The confidence coefficient Cronbach’s alpha was 0.71 in the previous study [22] and 0.89 in this study.

#### 2.3.2. Critical Thinking Disposition

The scale used in this study was the critical thinking disposition scale for nursing students developed by Kwon et al. [13]. It consists of a total of 35 questions, with questions 11, 12, 13, 14, 15, 16, 20, 22, and 26 being reverse scored. Each item is measured on a 5-point Likert scale from 1 (completely disagree) to 5 (completely agree). The higher the score, the higher the critical thinking tendency. Cronbach’s alpha was 0.89 at the time of development [13] and 0.81 in this study.

#### 2.3.3. Cognitive Ability

The scale used in this study was the cognitive ability scale developed by Lee et al. [1] to measure the cognitive ability of undergraduate students. This instrument consists of 34 items including knowledge, thinking, creativity, and problem-solving ability. Each item is measured on a 5-point Likert scale from 1 (completely disagree) to 5 (completely agree). The higher the score, the higher the cognitive ability. Cronbach’s alpha was 0.89 in the scale development study [1] and is 0.88 in this study.

#### 2.3.4. Learning Motivation

The scale used in this study was the learning motivation scale developed by Lee et al. [1] to measure the learning motivation of undergraduate students. It consists of 32 questions that measure emotion and motivation, and each item is measured on a 5-point Likert scale from 1 (completely disagree) to 5 (completely agree). The higher the score, the higher the motivation for learning. Cronbach’s alpha was 0.76 in the scale development study [1] and is 0.94 in this study.

#### 2.3.5. Learning Behavior

The scale used in this study was the learning behavior scale developed by Lee et al. [1] to measure the learning behavior of undergraduate students. It consists of 35 questions that measure learning behavior inside and outside the class, and each item is measured on a 5-point Likert scale. Lee et al. [1] reported a Cronbach’s alpha of 0.90. Cronbach’s alpha in this study is 0.80.

### 2.4. Data Collection and Ethical Considerations

After obtaining approval from the W University Institutional Review Board (IRB No. WKIRB-201703-SB-014), this study conducted a self-reported questionnaire survey from October to November 2017. Data were collected from undergraduate students who did not take classes established by this researcher so as to avoid any conflicts of interest. Prior to the survey, oral and written explanations were provided about the study background and purpose, the period of participation in this study, the procedure and method, the benefits and risks of participation, personal information protection, compensation for any losses due to participation, and withdrawal of consent. Only those subjects who voluntarily agreed to participate in this study filled out a written consent form. The subjects completed the structured questionnaire by themselves, and the responses were collected immediately. It took an average of 10 min to respond to the questionnaire, and a small gift was provided to the respondents.

### 2.5. Data Analysis

The collected data were analyzed using the SPSS 22.0 program (IBM Inc., Chicago, IL, USA). The general characteristics of the participants and the degree of helicopter parenting, critical thinking disposition, cognitive ability, learning motivation, and learning behavior were assessed using frequency, percentage, mean, and standard deviation. Pearson correlation coefficients were used to analyze the correlation between helicopter parenting, critical thinking disposition, cognitive ability, learning motivation, and learning behavior. Hierarchical multiple regression was used to identify the factors affecting learning behavior.

## 3. Results

### 3.1. General Characteristics of Participants

Table 1 presents the general characteristics of the participants in this study. The average age was 20.54 ± 2.19 years and 85.9% were female students. The degree of satisfaction with their major was 3.38 ± 0.81 points on average out of 5 points. The motive for choosing a nursing major was employment (51.7%), personal aptitude (22.1%), and acquaintance’s recommendation (19.5%).

### 3.2. Degree of Helicopter Parenting, Critical Thinking Disposition, Cognitive Ability, Learning Motivation, and Learning Behavior

Table 2 presents the average and standard deviation values of major variables. The helicopter parenting perceived by the participants was 3.06 ± 0.65, and the critical thinking disposition was 3.22 ± 0.32. The cognitive ability was 3.02 ± 0.47, and learning motivation was 3.08 ± 0.60. Finally, the learning behavior was 3.19 ± 0.36.

### 3.3. Correlation between Helicopter Parenting, Critical Thinking Disposition, Cognitive Ability, Learning Motivation, and Learning Behavior

Table 3 presents the correlation between major variables. Helicopter parenting showed a significant positive correlation with critical thinking disposition (r = 0.25, *p* = 0.002) and learning motivation (r = 0.17, *p* = 0.039). Learning behavior had a significant positive correlation with critical thinking disposition (r = 0.40, *p* < 0.001), cognitive ability (r = 0.29, *p* < 0.001), and learning motivation (r = 0.42, *p* = 0.004).

### 3.4. Influence of Helicopter Parenting, Critical Thinking Disposition, Cognitive Ability, and Learning Motivation on Learning Behavior

A hierarchical multiple regression analysis was conducted to verify the optimal model by checking whether the explanatory power of influence on learning behavior was increased. Helicopter parenting and critical thinking disposition were entered in Model Ⅰ, and helicopter parenting, critical thinking disposition, cognitive ability, and learning motivation were entered in Model Ⅱ (Table 4).

First, the Durbin–Watson statistic was used to check for autocorrelation of errors. The value was 1.50, which was close to 2.0, ensuring independence of the residuals. Second, to check for multicollinearity, the tolerance limit and variance inflation factor (VIF) were used. The tolerance limit was 0.80~0.94, which was higher than 0.10, and the VIF was 1.07~1.25, which was lower than 10. Therefore, it was confirmed that there was no problem of multicollinearity. Finally, a case-by-case diagnosis and the use of the Cook’s D statistic revealed that there were no outliers. Therefore, it was found that all the assumptions for the regression analysis were satisfied.

Model 1, which verified the effect of helicopter parenting and critical thinking disposition on learning behavior, showed 15% explanatory power. The analysis showed that the effect of helicopter parenting on learning behavior was not significant, but critical thinking disposition had a significant effect on learning behavior (β = 0.41, *p* < 0.001).

Model 2 verified the effect of helicopter parenting, critical thinking disposition, cognitive ability, and learning motivation on learning behavior. It showed an explanatory power of 32%, which was higher than that of Model 1. The analysis showed that the effect of helicopter parenting on learning behavior was not significant. However, learning motivation (β = 0.40, *p* < 0.001), cognitive ability (β = 0.26, *p* = 0.001), and critical thinking disposition (β = 0.25, *p* = 0.001) were shown to significantly affect learning behavior.

## 4. Discussion

This study was conducted to identify the factors affecting the learning behavior of nursing students. To this end, the level of helicopter parenting, critical thinking disposition, cognitive ability, learning motivation, and learning behavior of nursing students were analyzed. The discussion based on the main results of this study is as follows:

The degree of helicopter parenting perceived by the participants was three points on average, which could be interpreted as a low level. This result was similar to that reported in a study by Cho and Yim [23], which analyzed helicopter parenting by parents of nursing students. It was also similar to the findings of a study by Kim and Park [2], which reported that college students’ perceived degree of the parenting attitude of parents was low. Choi [10] reported that Korean students have ambivalent perceptions with respect to positively recognizing parental care, while they recognize the problems of helicopter parenting. In the highly competitive and achievement-oriented Korean social culture, undergraduate students who positively recognize parental overprotection may have low sensitivity to helicopter parenting. For this reason, it may be interpreted that the nursing students’ perceived level of helicopter parenting was low. In addition, since this study was limited to Chungcheong Province and Jeolla Province, while not including Seoul and other metropolitan areas, where parents’ academic enthusiasm is high, this result may reflect regional characteristics. As problems related to helicopter parenting are constantly emerging internationally, it is necessary to continuously and repeatedly analyze helicopter parenting of nursing students.

The degree of critical thinking disposition of nursing students was three points on average in this study, which could be interpreted as a moderate level. This is similar to findings of previous studies [14,15,28], which reported that nursing students’ average level of critical thinking disposition was three out of a total of five points. Critical thinking is a concept suggested in the standards for accreditation of the Korean nursing curriculum, and is actually one of the concepts used by many nursing colleges as educational outcomes [29,30]. While critical thinking is an important competency for nursing students, the level of critical thinking disposition of nursing students is insufficient in reality. Yang and Sim [14] reported that nursing students who chose their major based on their aptitude or who were highly satisfied with their major had a high tendency to think critically. In this study, only 22.1% of the participants chose their major in consideration of their aptitude, while 71.2% of the participants chose nursing as their major considering employment opportunities or due to the recommendation of an acquaintance. In addition, the degree of perceived satisfaction with their major was moderate. In this study, the level of critical thinking disposition was not good because it reflects the general characteristics of the participants in this study.

Nursing students’ level of cognitive abilities was three points on average, which can be interpreted as being at a medium level. This was consistent with the results of a study by Lee et al. [1], which analyzed the learning competencies of college students and reported that their cognitive abilities were at a moderate level. Cognitive ability includes thinking ability to analyze and criticize, as well as creativity and problem-solving ability [1]. In-depth comparative analysis was not possible because there were not enough previous studies that analyzed the cognitive ability of nursing students. However, the results of this study were similar to those of previous studies that reported that nursing students’ metacognition, a concept similar to cognitive ability, was at a moderate level [28,31]. In order to develop competent nurses with the ability to adapt to the rapidly changing healthcare environment, problem-solving skills are being treated as an important component of the nursing educational curriculum [2,3]. It was found that the cognitive ability of the participants in this study was not high. Therefore, nursing education should aim to improve cognitive ability by applying various teaching methods with a focus on cognitive ability to help students analyze and solve problems.

The degree of learning motivation of the participants was three points on average, which can be interpreted as a medium level. The learning motivation of nursing students reported in previous research was moderate and similar to the results of this study [18,24,25]. Learning motivation includes self-determination, learning goal orientation, and self-efficacy [1]. Most of the participants chose nursing as their major without considering their aptitude, and their satisfaction with their major was also moderate in this study. This characteristic is thought to have had an effect on lowering the motivation to learn nursing. The motivation for learning also includes emotional factors such as test anxiety and learning stress [1]. It has been reported that nursing students always experience as much anxiety before a test as college students of other majors [32]. For this reason, it can be interpreted that the level of motivation for learning was not high among the nursing students in this study.

The subjects’ learning behavior in this study was an average of three points, which was a medium level. There is a limitation in comparative discussion due to the lack of prior studies that analyzed learning behavior in nursing college students. Compared with previous studies that reported that general undergraduate students’ average learning behavior scores were 3.7 or more out of five [1,33], the level of learning behavior was somewhat insufficient among this study’s participants. While the preceding studies involved university students from all years, this study’s result is thought to reflect the characteristics of its participants, who were all second-year undergraduate students. Learning behaviors include effective concentration and memory strategies, notebook organization, effort and learning environment control, resource utilization, and career preparation [1]. In other words, it is possible that sophomore students have not yet acquired effective learning behaviors in nursing. Therefore, for effective learning, it is necessary to use strategies such as mentoring of nursing students to find a learning method that suits them.

As a result of analyzing the factors influencing the learning behavior of nursing students, it was found that learning motivation has the greatest effect on learning behavior. This result was supported by a study by Lee and Lee [33], which found that it is necessary to manage learning motivation in order to promote the academic behavior of college students because learning motivation has a significant influence on their academic behavior. In particular, the nursing educational curriculum provides both theoretical and practical education, so the academic burden is heavy [2,3]. In addition, as shown in this study, there may be a lack of motivation for learning because the choice of a nursing major occurs due to factors other than interest in or aptitude for nursing. Therefore, it is necessary to improve the learning motivation of nursing students for effective learning. In particular, as a result of analyzing factors influencing learning behavior in this study, it was found that the explanatory power of the model including cognitive ability and learning motivation was higher. Therefore, when dealing with the learning behavior of nursing students, cognitive ability and learning motivation should be considered together.

On the other hand, it was found that helicopter parenting did not have a significant effect on the learning behavior of nursing students. LeMoyne and Buchanan [34] reported that helicopter parenting could be a factor that hinders children’s learning and competitiveness. Several previous studies have emphasized that undergraduate students raised by helicopter parents may have low self-esteem or self-efficacy due to excessive parental control and bondage [20,21,22]. In addition, previous studies have also reported that the parenting behavior of helicopter parents is related to academic burnout and negatively affects the academic achievement of undergraduate students [6,7]. This study found that although helicopter parenting did not have a significant direct effect on learning behavior, it was significantly correlated with critical thinking disposition and learning motivation. Therefore, it is necessary to verify its indirect effect on learning behavior. There is a limit to the generalizability of the results of this study due to insufficient analysis of helicopter parenting for nursing students.

This study is meaningful in that it attempted to analyze and confirm helicopter parenting, which is an emerging concept, for nursing students. In addition, it confirmed basic learning competencies for nursing students because it dealt with the cognitive abilities, learning motivations, and learning behaviors that undergraduate students must have. On the other hand, this study has limitations in terms of generalizing the results to international nursing students because the data were collected using convenience sampling for second-year nursing students. Since helicopter parenting is a recent issue, there are not many prior studies related to this. Therefore, there were limitations in discussing the results of this study. It is necessary to make an effort to generalize helicopter parenting for nursing students through repeated verification of parenting of helicopter parents.

## 5. Conclusions

This study involved a descriptive survey of 149 nursing students to investigate the effects of helicopter parenting, critical thinking disposition, cognitive ability, and learning motivation on learning behavior. First, nursing students’ critical thinking disposition, cognitive abilities, learning motivations, and learning behaviors were found to be at moderate levels. Therefore, nursing education must develop effective teaching methods to cultivate their critical thinking tendencies and learning competency. Second, learning motivation was found to be the most influential factor for learning behavior. Therefore, learning motivation of nursing students should be considered important for effective learning. Finally, nursing students’ learning motivation was not satisfactory. Therefore, it is necessary to develop and verify effective strategies that can induce learning motivation in nursing students.

Based on this study, the following suggestions are made. Further research is needed to analyze helicopter parenting, cognitive ability, learning motivation, and learning behavior of nursing students from multiple angles. Additionally, it is suggested that data be gathered to generalize the results by conducting an expanded study involving not only second-year students, but also freshmen and third- and fourth-year students in the Department of Nursing. This study found that helicopter parenting did not affect the learning behavior of nursing students, which is thought to reflect the collected data because this study was limited to certain areas. Therefore, it is suggested that the finding be verified by conducting further studies in metropolitan areas, where there is high academic enthusiasm.

## Figures and Tables

**Table 1:** General characteristics of participants (*n* = 149).

Characteristic	Category	*n* (%)/Mean ± SD
Age (years)		20.54 ± 2.19
Gender	Male	21 (14.1)
	Female	128 (85.9)
Satisfaction of major		3.38 ± 0.81
Academic performance	≥ 4.0	18 (12.1)
	≤ 3.5–<0 4.0	52 (34.9)
	≤ 3.0–<0 3.5	59 (39.6)
	<0 3.0	20 (13.4)
Motivation of majoring	Employment	77 (51.7)
	Aptitude	33 (22.1)
	Acquaintance’s recommendation	29 (19.5)
	Others	10 (6.7)

**Table 2:** Levels of helicopter parenting, critical thinking disposition, cognitive ability, learning motivation, and learning behavior (*n* = 149).

Variable	Mean ± SD
Helicopter parenting	3.06 ± 0.65
Critical thinking disposition	3.22 ± 0.32
Cognitive ability	3.02 ± 0.47
Learning motivation	3.08 ± 0.60
Learning behavior	3.19 ± 0.36

**Table 3:** Correlation of helicopter parenting, critical thinking disposition, cognitive ability, learning motivation, and learning behavior (*n* = 149).

Variable	Helicopter Parenting	Critical Thinking Disposition	Cognitive Ability	Learning Motivation
	r (*p*)	r (*p*)	r (*p*)	r (*p*)
Critical thinking disposition	0.25 (0.002)			
Cognitive ability	−0.05 (0.542)	0.28 (0.001)		
Learning motivation	0.17 (0.039)	0.24 (0.004)	−0.10 (0.217)	
Learning behavior	0.04 (0.601)	0.40 (<0.001)	0.29 (<0.001)	0.42 (0.004)

**Table 4:** Factors affecting learning behavior among participants (*n* = 149).

Variables	Model I					Model II				
	B	SE	β	t	*p*	B	SE	β	t	*p*
(Constant)	1.78	0.29		6.23	<0.001	1.07	0.29		3.75	<0.001
Helicopter parenting	−0.03	0.04	−0.06	−0.78	0.438	−0.04	0.04	−0.08	−1.05	0.295
Critical thinking disposition	0.47	0.09	0.41	5.25	<0.001	0.29	0.09	0.25	3.30	0.001
Cognitive ability						0.20	0.06	0.26	3.53	0.001
Learning motivation						0.24	0.04	0.40	5.58	<0.001
	Adj. R^2^ = 0.15, F = 13.93, *p* < 0.001					Adj. R^2^ = 0.32, F = 18.21, *p* < 0.001				

Adj. = adjusted, SE = Standard Error.

## Data Availability

The data presented in this study are available on request from the corresponding author.
